# In Vitro Evaluation of Electrospun PCL Bioscaffold with Zinc-Doped Bioactive Glass Powder Addition

**DOI:** 10.3390/polym16192811

**Published:** 2024-10-04

**Authors:** Ya-Yi Chen, Yuh-Jing Chiou, Pei-Jung Chang, Wei-Min Chang, Yu-Cheng Yeh, Chin-Yi Chen, Yu-Kang Chang, Chung-Kwei Lin

**Affiliations:** 1 Doctoral Program in Medical Biotechnology, National Chung Hsing University, Taichung 402, Taiwan; yayichen2013@gmail.com; 2 Department of Stomatology, Tung’s Taichung Metro Harbor Hospital, Taichung 435, Taiwan; 3 Research Center of Digital Oral Science and Technology, College of Oral Medicine, Taipei Medical University, Taipei 110, Taiwan; chiou@gm.ttu.edu.tw (Y.-J.C.); weiminchang@tmu.edu.tw (W.-M.C.); chencyi@fcu.edu.tw (C.-Y.C.); 4 Department of Chemical Engineering and Biotechnology, Tatung University, Taipei 104, Taiwan; 5 Graduate Institute of Manufacturing Technology, National Taipei University of Technology, Taipei 106, Taiwan; 6 School of Oral Hygiene, College of Oral Medicine, Taipei Medical University, Taipei 110, Taiwan; 7 Department of Materials Science and Engineering, Feng Chia University, Taichung 407, Taiwan; ucyeh0910@gmail.com; 8 Department of Medical Research, Tung’s Taichung Metro Harbor Hospital, Taichung 435, Taiwan; 9 Department of Post-Baccalaureate Medicine, College of Medicine, National Chung Hsing University, Taichung 402, Taiwan; 10 Department of Nursing, Jenteh Junior College of Medicine, Nursing and Management, Miaoli 356, Taiwan; 11 School of Dental Technology, College of Oral Medicine, Taipei Medical University, Taipei 110, Taiwan; 12 Additive Manufacturing Center for Mass Customization Production, National Taipei University of Technology, Taipei 106, Taiwan

**Keywords:** PCL, zinc-doped bioactive glass, electrospinning, bioscaffold, bioactivity, antibacterial performance

## Abstract

Preparing electrospun fibers by applying a potential difference between a polymeric solution and a contacting substrate is increasingly attracting attention in tissue engineering applications. Among the numerous polymers, polycaprolactone (PCL) bioscaffold has been widely investigated due to its biocompatibility and biodegradability. Bioactive powder can be added to further improve its performance. In the present study, bioactive glass powder modified by adding 0–6 wt.% antibacterial zinc element (coded as ZBG) was prepared through the sol–gel process. Furthermore, PCL bioscaffolds with various ZBG additions were prepared using the electrospinning technique. The zinc-doped bioactive glass powder and electrospun PCL/ZBG bioscaffolds were evaluated using scanning electron microscopy, X-ray diffraction and Fourier-transform infrared spectroscopy to determine their structural properties. Additionally, in vitro bioactivity, biocompatibility and antibacterial performance were investigated. Experimental results showed that sol–gelled ZBG powder possessed superior bioactivity and 0.8 g ZBG was the optimal addition to prepare PCL/ZBG bioscaffolds with. All the electrospun PCL/ZBG bioscaffolds were biocompatible and their antibacterial performance against two *S. aureus* strains (SA133 and Newman) improved with increasing zinc concentration. Electrospun PCL/ZBG bioscaffolds exhibited excellent bioactivity and have great potential for biomedical application.

## 1. Introduction

Electrospinning is a technology commonly used to prepare fibers with a diameter of a few hundred nanometers. By applying a potential between the precursor polymeric solution and the contacting substrate, electrospun fibers can be collected. Experimental parameters affecting the properties of electrospun fibers may include the processing parameters of the equipment (the size and shape of the injection needle, applied voltage, feed rate, working distance, etc.), the polymeric solution (type of polymer, concentration, molecular weight, viscosity, conductivity, etc.) and the ambient environment (temperature and humidity). Excellent reviews that focus on various topics concerning electrospinning are available [1,2,3,4]. For instance, Maji and Pramanik compared the electrospinning techniques, summarized their process parameters and their development and limitation [1]. Sun at al. reviewed the electrospinning factors, the types of electrospinning and their applications in drug-controlled release, biological dressings, tissue repair and enzyme immobilization [2].

Among the numerous polymers used in electrospinning is polycaprolactone (PCL), a biocompatible and biodegradable polymeric material that has attracted increasing R and D attentions [5,6]. Electrospun PCL fibers and bioscaffolds have been widely investigated. For example, different inorganic additives [7,8,9] and various solvents [5,6,10] have been used to prepare electrospun PCL-based scaffolds. For another example, Abel at al. discussed the effect of benign solvents (i.e., acetic, formic acids, water and their mixtures) and standard organic solvents (i.e., chloroform) for electrospinning [11]. Banimohamad-Shotorbani et al. reviewed the usage of the nanofiber of PCL/HAp for scaffold improvement [8]. Pedrosa et al. investigated polycaprolactone (PCL) membranes associated with 0–10 wt.% nanocrystalline hydroxyapatite (HA) and 5 wt.% Zn-doped HA (ZnHA) where HA (or ZnHA) served as a nucleating agent and thermal stabilizer [12]. Saiding and Cui reported that organic, inorganic and mesoporous nanoparticles combined with electrospun nanofibers can be applied for drug delivery and regenerative medicine [9]. Ismail et al. studied a 3D elastomeric fibrous mesh for cardiac tissue engineering applications. They prepared acrylated poly(1,10-decanediol-co-tricarballylate) copolymer by utilizing the photoreactive electrospinning process with ultraviolet radiation for crosslinking. The study showed a significant increase in cell attachment and growth on the electrospun fibers compared to the reference [13].

Bioactive glass (i.e., BG) possesses superior biocompatibility, making it a promising material for biomedical application as an inorganic powder additive for bioscaffolds. BG with a wide range of compositions have been investigated, starting from 45S [14] up to 80S [15] with 45 and 80 wt.% SiO_2_, respectively. In order to expand the applications of BG powder, modification through the introduction of surfactants during the process to increase the specific surface area [15] or by replacing calcium with other elements for specific biological functionalities [16] have been attempted. For instance, Liverani et al. studied the addition of BG particles to PCL-based polymer electrospun fibers using benign solvents [17] and the enhancement of shape memory properties without the need of thermal crosslinking treatment [18]. During biomedical applications of bioscaffolds, the local tissue inflammation induced by *Staphylococcus aureus* (*S. aureus*) is a critical issue, due to the bacteria destroying the osseointegration of orthopedics and dental implants [19,20]. In addition, pathogenic infections in hospital and healthcare treatments are commonly attributed to the presence of *S. aureus* [21], which is the cause of 34% of all biomaterial-associated infections [22]. Meanwhile, SA113 and Newman are both potently virulent strains that cause severe tissue inflammation and form biofilm to resist antibiotic treatment [23,24]. Thus, novel antibacterial processing and material modification have been attempted to further extend the potential applications of BG-added bioscaffolds [25].

As mentioned above, various investigations concerning PCL-based bioscaffolds have been attempted. A comprehensive study concerning the synthesis, biocompatibility, bioactivity and antibacterial performance of PCL bioscaffold prepared by using benign solvents with different amounts of Zn-doped bioactive glass was lacking. In the present study, bioactive glass with 58 wt.% SiO_2_ (58S) was prepared using the sol–gel process. Zinc elements (0–6 wt.%) were introduced into the bioactive glass to add to their antibacterial ability. An optimal amount of zinc-doped bioactive glass powder (ZBG) was incorporated into the PCL solution for electrospinning. The in vitro evaluations of the bioactivity, biocompatibility and antibacterial performance of these electrospun PCL/ZBG composite bioscaffolds were investigated to determine their feasibility and potential for tissue engineering applications.

## 2. Materials and Methods

### 2.1. Preparation and Characterization of ZBG Powder

The bioactive glass is composed of 58 wt.% SiO_2_, 33 wt.% CaO and 9 wt.% P_2_O_5_. Part of CaO was replaced by ZnO (0, 2, 4 and 6 wt.%, coded as 0ZBG to 6ZBG, respectively) to prepare zinc-doped bioactive glass powder. Nitric acid (1 M, 5 mL) was first mixed with 15 mL deionized water and, subsequently, 5 mL of tetraethoxysilane (TEOS; SIGMA, St. Louis, MO, USA) and 0.5 mL of triethyl phosphate (TEP; SIGMA, St. Louis, MO, USA) and the designed composition of calcium nitrate tetrahydrate (Ca(NO_3_)_2_·4H_2_O; SIGMA, St. Louis, MO, USA) and Zn(NO_3_)_2_·6H_2_O; J.T. Baker, Radnor, PA, USA) were added into the solution. After aging, the obtained gels were dried at 80 °C for 24 h, followed by calcination at 700 °C (determined by the following thermogravimetric analysis) for 3 h. Table 1 summarizes the corresponding sample codes for the 700 °C-calcined zinc-added bioactive glass powder used in the present study.

A simultaneous DSC-TGA instrument (SDT2960, TA Instruments, New Castle, DE, USA) was used to measure the heat flows (Differential Scanning Calorimetry, DSC) and weight changes (Thermogravimetric Analysis, TGA) of 30 mg dry gelled powder within temperatures ranging from 25 to 900 °C with a heating and cooling rate of 10◦C/min under ambient atmosphere to determine the calcination temperature. After calcination, the ZBG powder (0–6 wt.% zinc addition) was examined using field emission scanning electron microscopy (FE-SEM, S-4800, Hitachi, Tokyo, Japan) to observe the powder morphology with an imaging condition using 3 kV electron landing energy at 4 mm working distance. An X-ray diffractometer (D8 Discover, Bruker, Billerica, MA, USA) was used to examine the crystalline structure using Cu Kα radiation (λ = 1.542 Å) with a scan rate of 2°/min in the 20–80° 2θ range with a LynxEye detector. Fourier-transform infrared spectroscopy (FTIR spectrometer Frontier, Perkin Elmer, Waltham, MA, USA) was used to determine the binding energies of various functional groups via the attenuated total reflectance (ATR) mode within a wavelength of 600–4000 cm^−1^. The bioactivity of the ZBG powder was evaluated by immersing them into simulated body fluid (SBF) with a ratio of ZBG:SBF = 0.2 g:80 mL. The desired SBF was prepared according to the literature [26]. The immersed powder was then cleaned with deionized water and dried at 80 °C. The ZBG powder after SBF immersion was examined using FE-SEM, XRD and FTIR as addressed above.

### 2.2. Electrospinning and Characterization of Electrospun ZBG/PCL Bioscaffolds

The colloidal precursor solution for electrospinning was prepared by adding 1.2 g of PCL (product number: 440744, melting point: 60 °C, beads: ~3 mm, density: 1.145 g/mL at 25 °C, M_n_ 80,000, SIGMA, St. Louis, MO, USA) to 3 mL of acetic acid and 7 mL of formic acid. Various amounts of 0ZBG (0.6, 0.8 and 1.0 g) were added to the precursor solutions. Electrospinning (FES-COS; Falco Tech Enterprise Co., Ltd., New Tiapei City, Taiwan) was used to prepare the PCL and the PCL/ZBG scaffolds on a titanium substrate [27]. The electrospinning parameters follow the work by Liverani et al. [17] and were adjusted as follows: pump flow rate of 0.5 mL per hour, voltage of 12 kV, working distance of 12 cm, spinning for 1 h and drying at room temperature for 12 h. FE-SEM images were then analyzed using Image J software (version 1.54b, Wayne Rasband, National Institutes of Health, Bethesda, MD, USA) to determine the diameter distributions of the electrospun fibers (~100 measurements) and the optimal amount of ZBG addition (0.8 g in the present study). The electrospun PCL/ZBG bioscaffolds with 0.8 g 0–6ZBG additions were coded as ES-0ZBG and ES-6ZBG, respectively. A bioactivity evaluation was performed by immersing the selected ES-ZBG bioscaffold in SBF solution. An FE-SEM observation and an X-ray diffraction were used to examine the hydroxyl apatite formation. In addition, Inductively Coupled Plasma-Optical Emission Spectrometry (725 ICP-OES, Agilent, Santa Clara, CA, USA) was used to determine the Zn^2+^ concentration within the residue SBF solution after various immersion durations.

The biocompatibility of the ES-ZBG bioscaffold was evaluated by determining the mitochondrial dehydrogenase activity using the Cell Counting Kit-8 (CCK-8) assay (Dojindo, Japan). Three different concentrations of extracts (200, 20 and 2 mg/L) with or without antibiotic ES-ZBG fibers were performed using the Dulbecco’s Modified Eagle Medium (DMEM) extraction for 24 h in 1.5 mL microcentrifuge tubes (Gunster Biotech, New Taipei City, Taiwan) and the extracts were sterilized via 0.22 μm pore-size filtration. 5 × 10^4^ L929 murine normal fibroblasts (suggested by ISO standard) were used to test the biocompatibility of the ES-ZBG bioscaffolds. To determine long-term biocompatibility with ZBG promoting biological function, MG-63 human osteoblast-like osteosarcoma cells were seeded on 96 well plates and incubated with different ZBG extracts for 1, 3 and 7 days. The testing cells were maintained at 37 °C in a humidified atmosphere containing 5% CO_2_ condition. At the endpoint, the medium was replaced by a fresh medium with 10% CCK-8-containing medium and incubated for an additional 4 h. The biocompatibility was measured at OD 450 nm using a Multiskan™ FC Microplate Photometer (Thermo-Fisher, Waltham, MA, USA).

The antibacterial performance of ES-ZBG bioscaffold was investigated using an antibacterial disk diffusion assay and repeated three times. The different content of the ZBG fibers were shaped into 10 mm discs by mold. The positive control was a disk soaked with 30 μg kanamycin and the negative control was ES-0ZBG without kanamycin. Two *Staphylococcus aureus* bacteria strains (SA133 and Newman) were isolated from the tubercular osteomyelitis, cultured in brain–heart infusion (BHI) broth, diluted to MacFarlane 0.5, swapped on a BHI broth agar plate and challenged with an ES-ZBG disc at 37 °C for 16 h. Thereafter, the mean diameter of the inhibition zone was measured [28].

## 3. Results and Discussion

In the present study, zinc-doped bioactive glass (i.e., ZBG) was first prepared via the sol–gel process. Characterization of the original and calcined ZBG was performed to determine the materials’ properties and biocompatibility. Optimal ZBG powder was added into the PCL for electrospinning. Electrospun ZBG/PCL bioscaffolds were then investigated in vitro to elucidate their biocompatibility and antibacterial performance.

### 3.1. Characteristics of ZBG Powder

**Figure 1:**
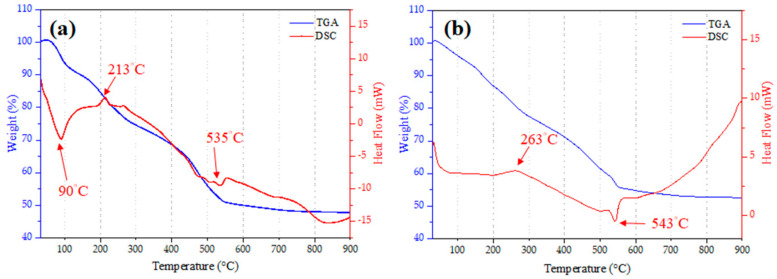
TGA and DSC curves of (**a**) 0ZBG and (**b**) 6ZBG powders.

Figure 1 shows the TGA and DSC curves of 0ZBG and 6ZBG powder. As shown by the TGA curve (the blue line) of 0ZBG powder (Figure 1a), apparent weight loss can be observed with increasing temperatures. Basically, it can be roughly divided into two regions according to the slope of the TGA curve. An obviously continuous decrease in weight (49.0%) can be observed from room temperature up to 535 °C, whereas only 3.3% weight loss was exhibited thereafter. The transition can be better revealed by the DSC curve (the red line) in Figure 1a. An endothermic peak caused by residual water was observed at 90 °C. An exothermic peak was observed at 213 °C that can be attributed to the decomposition of the functional groups used during the process. Another endothermic peak at 535 °C was perceived due to the removal of nitrate. This shows a similar trend as reported in the literature [29,30]. Figure 1b shows the TGA and DSC curves for 6 wt.% zinc added in bioactive glass powder (6ZBG). The corresponding weight losses at the first and second regions were respectively 44.5 and 2.9 wt.% and were slightly smaller than those of 0ZBG powder. The endothermic peak at 90 °C, however, was almost absent. This was probably due to the relatively small amount of physically absorbed water in 6ZBG (~3.8%, weight loss up to 100 °C) compared to that of 0ZBG (6.5%) and the presence of Zn ions that hindered the water decomposition. The subsequent exothermic and endothermic peaks shifted to 263 and 543 °C, respectively. The addition of zinc slightly delayed the reaction to a higher temperature, but basically resulted in a behavior similar to that of 0ZBG. As revealed by TGA results, limited weight loss (less than 1 wt.%) can be observed after 700 °C and this temperature was used for the subsequent calcination of the sol–gelled powder.

**Figure 2:**
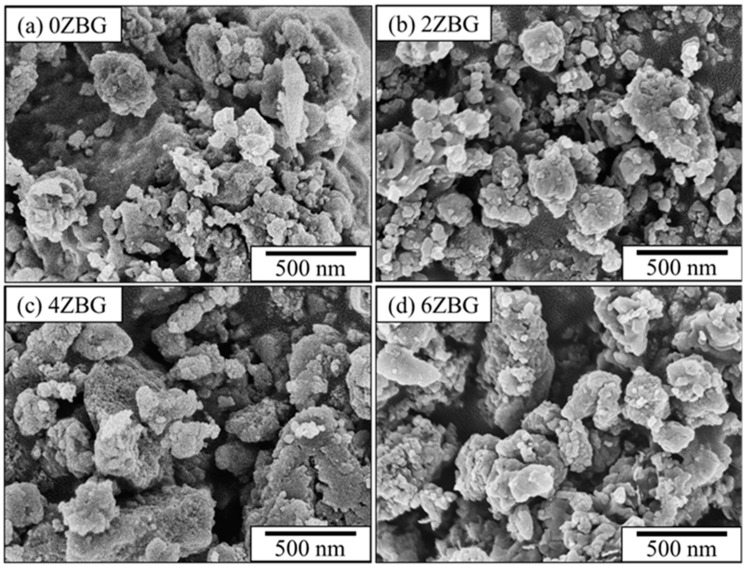
SEM images of (**a**) 0ZBG, (**b**) 2ZBG, (**c**) 4ZBG and (**d**) 6ZBG powders.

**Figure 3:**
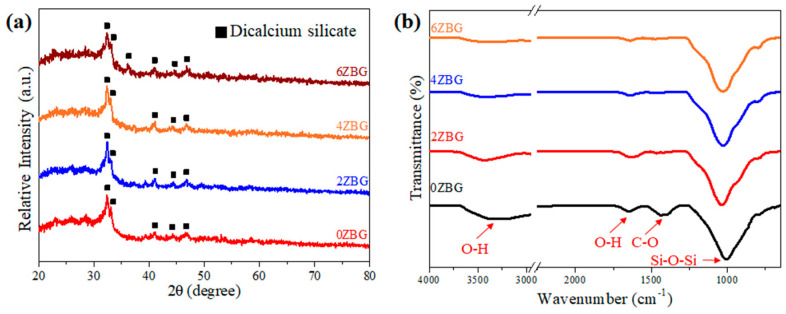
(**a**) XRD patterns and (**b**) FTIR spectra for 0ZBG-6ZBG powders.

Figure 2 shows the SEM images of the BG and ZBG powder that was prepared via the sol–gel process, followed by its calcination at 700 °C for 3 h. Severe agglomeration can be observed for all the powders investigated in the present study. The agglomerated particles exhibited a particle size less than 1 μm. The crystalline structure of the ZBG powder was examined using XRD and FTIR techniques. Figure 3a shows the XRD patterns of ZBG powder that exhibited crystalline peaks of dicalcium silicate (β-Ca_2_SiO_4_, PDF No. 01-077-409) [10,31] with an amorphous phase background. Similar FTIR spectra were also observed for all the ZBG powders. The bands at 998, 1419 and 1635 cm^−1^ were attributed to Si-O-Si, C-O and O-H functional groups, respectively. In addition, there was a wide OH stretching vibration band around 3200–3600 cm^−1^.

**Figure 4:**
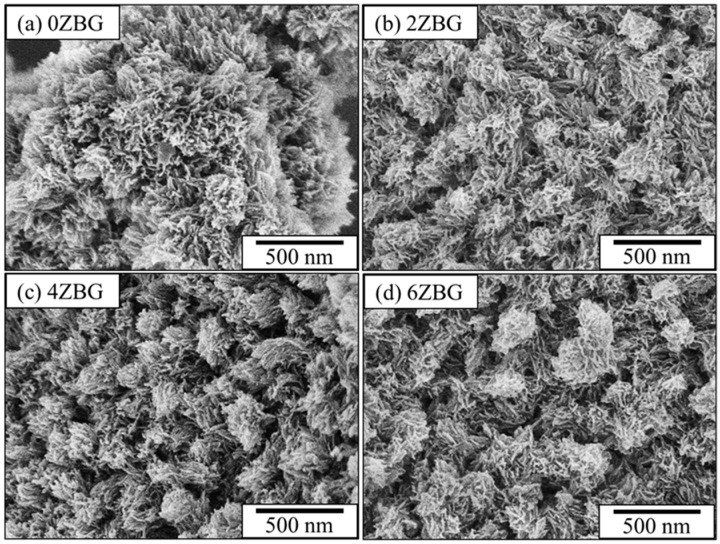
SEM images of (**a**) 0ZBG, (**b**) 2ZBG, (**c**) 4ZBG and (**d**) 6ZBG powders after immersion in SBF solution for 7 days.

**Figure 5:**
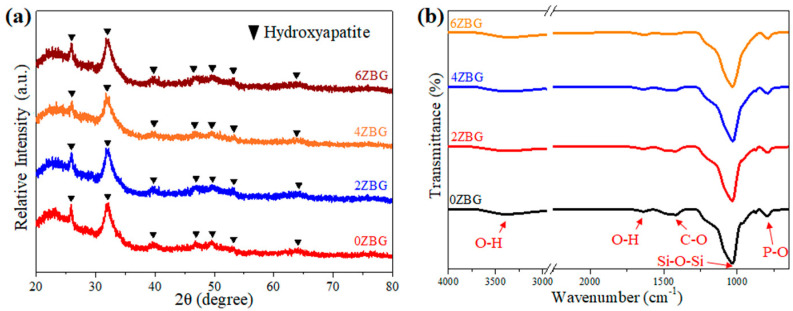
(**a**) XRD patterns and (**b**) FTIR spectra for 0ZBG-6ZBG powders after immersion in SBF solution for 7 days.

The bioactive performance of these ZBG powders was investigated by immersing them in simulated body fluid for 7 days. As shown in Figure 4, it can be noted that all the agglomerated ZBG powder formed a coral-like structure, probably due to the dissolution of bioactive glass and the formation of hydroxyl apatite. This suggests that the so-prepared ZBG powder exhibited superior bioactivity and shows a similar trend as reported in the literature [32]. The formation of hydroxyl apatite was confirmed by X-ray diffraction and FTIR spectroscopy. Figure 5a shows the XRD patterns of ZBG powder after its immersion in SBF solution for 7 days. Compared to those shown in Figure 3a, the diffraction peaks of dicalcium silicate were replaced by the diffraction peaks of hydroxyl apatite (PDF No. 01-1008). This indicated the formation of hydroxyl apatite. The obvious FTIR band at 796 cm^−1^ (Figure 5b) due to the P-O functional group also confirmed the existence of hydroxyl apatite.

### 3.2. Evaluation of Electrospun ZBG/PCL Bioscaffold

#### 3.2.1. Effect of ZBG Powder Addition

**Figure 6:**
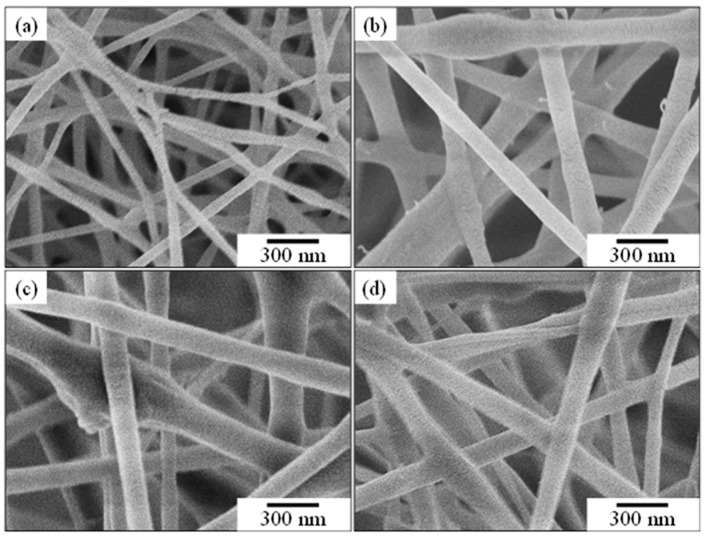
Electrospinning fibers with (**a**) 0, (**b**) 0.6, (**c**) 0.8 and (**d**) 1.0 g of bioactive glass powder additions.

**Figure 7:**
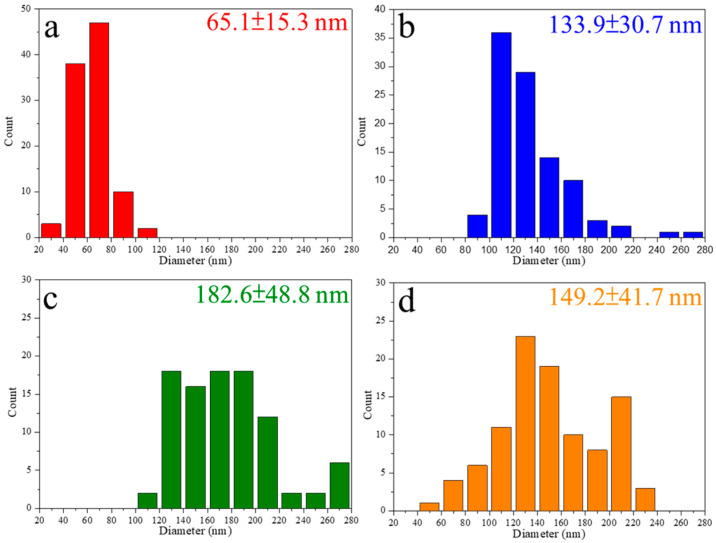
Diameter distributions of electrospun fibers with (**a**) 0, (**b**) 0.6, (**c**) 0.8 and (**d**) 1.0 g of bioactive glass powder additions.

Before electrospinning, it is important to determine the suitable amount of ZBG powder addition within the PCL solution. Figure 6 shows the SEM images of a series of electrospun fibers. Figure 6a shows the electrospun PCL fiber without ZBG addition. The average diameter of the PCL fiber, determined via the SEM images using an Image J software, was 65.1 ± 15.3 nm. After adding the ZBG powder, a significant increase in the diameter of electrospun PCL/ZBG fibers can be observed. As shown in Figure 6b–d, electrospun PCL/ZBG fibers with 0.6, 0.8 and 1.0 g ZBG addition had an average diameter of 133.9 ± 30.7 nm, 182.6 ± 48.8 nm and 149.2 ± 41.7 nm, respectively, as seen in Figure 7. The average diameter reached a maximum with 0.8 g of ZBG addition and decreased with 1.0 g addition. It should be pointed out that superfluous amounts of powder addition (i.e., 1.0 g) induced powder agglomeration and caused sedimentation. Consequently, the diameter of the electrospun PCL/ZBG fibers with 1.0 g ZBG were smaller than those with 0.8 g ZBG. Additionally, discontinuity due to agglomerated BG powder occurred during electrospinning. It is expected that the larger the amount of ZBG addition, the better the antibacterial performance of the bioscaffold. Thus, the electrospun PCL/ZBG fibers with 0.8 g powder addition were used for further experiments.

#### 3.2.2. In Vitro Investigation of Electrospun PCL/ZBG Bioscaffold

**Figure 8:**
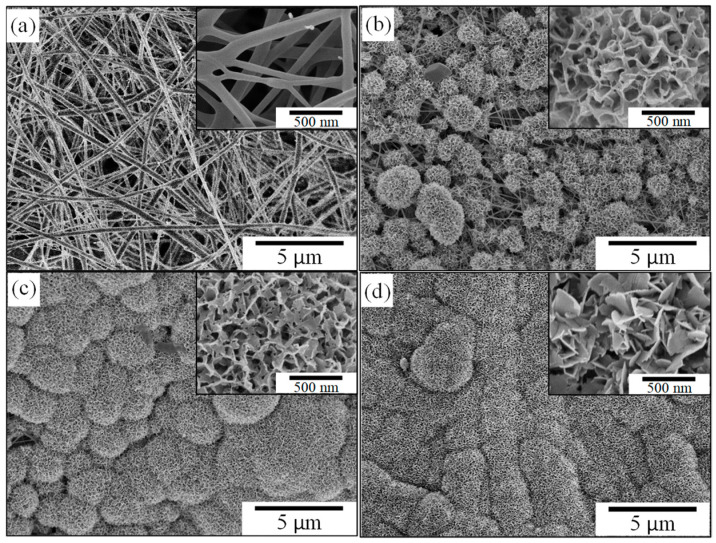
SEM images of ES-0ZBG bioscaffolds (**a**) before and after immersion in SBF solutions for (**b**) 1 day, (**c**) 3 days and (**d**) 7 days. Higher magnification SEM images were shown as the inserts on the upper-right corners.

Electrospun PCL/ZBG bioscaffolds with different amounts of zinc addition (0ZBG-6ZBG) looked similar and the major difference was the amount of zinc addition. Thus, ES-0ZBG bioscaffolds were used as a prototype and Figure 8 shows the SEM images of them before and after their immersion in SBF solution for various durations. Figure 8a shows the as-prepared ES-0ZBG bioscaffold at a relatively low magnification. The insert image used a higher magnification, but it was slightly lower than that shown in Figure 6c (please note the differences in scale bars). After immersion in SBF for 1 day, cauliflower-like particles formed on the electrospun PCL/ZBG fibers (Figure 8b). As shown in the insert image with a higher magnification, coral-like HA particles can be observed and are similar to those shown in Figure 4 for ZBG powder. As shown in Figure 8c, no electrospun fibers can be observed and the cauliflower-like particles grew larger after their immersion in SBF for 3 days. The continuous growth of the cauliflower-like particles and the diminishment of pores were observed after immersing them for 7 days, as seen in Figure 8d.

**Figure 9:**
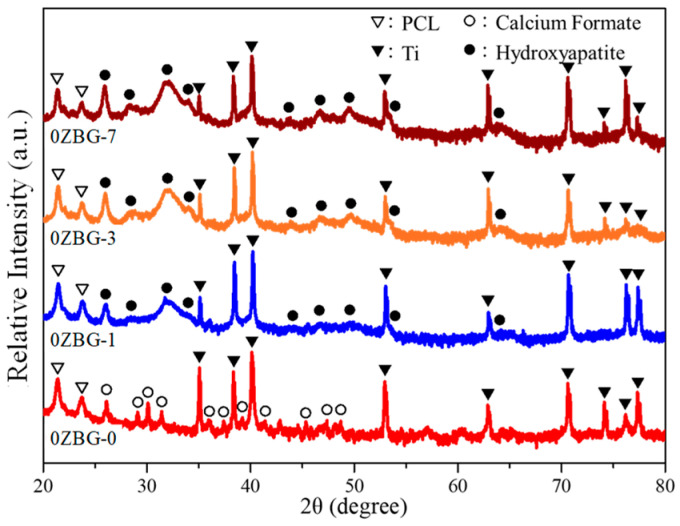
XRD patterns of ES-0ZBG fibers before and after immersion in SBF solutions for 1, 3 and 7 days.

In order to elucidate the structure of the precipitated particles after SBF immersion, X-ray diffraction examination was performed and Figure 9 shows the corresponding XRD patterns. It can be noted that, in addition to the diffraction peaks from the Ti substrate, PCL and calcium formate (PDF No. 26-0937) can be observed before immersion. The diffraction peaks of calcium formate disappeared after immersion and those of HA were exhibited. The intensity of the HA diffraction peaks increased with the increasing immersion time. The results indicated that the continuous formation of hydroxyl apatite occurred during the SBF immersion [33].

**Figure 10:**
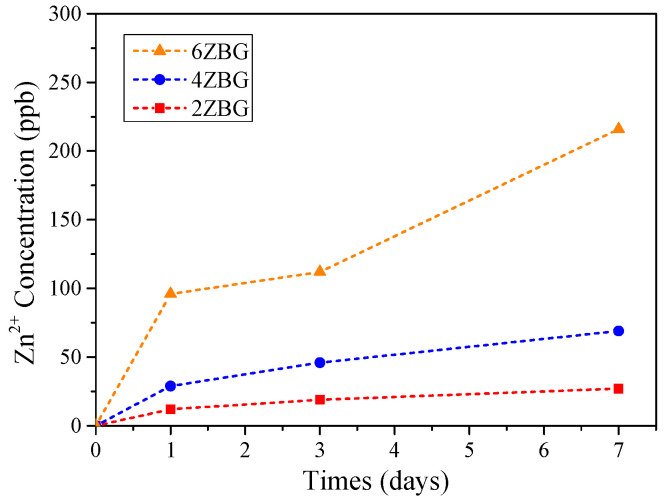
Zn^2+^ ion concentration of various ES-ZBG bioscaffolds after immersion in SBF solutions for 1, 3 and 7 days.

For the various electrospun PCL/ZBG bioscaffolds, the concentration of the Zn^2+^ ion will be an important issue for antibacterial performance [34]. As shown in Figure 10, it is obvious that the Zn^2+^ concentration increased not only with the Zn addition in ES-ZBG, but also with the immersion time. At the end of the 7 days of SBF immersion, the Zn^2+^ concentration was 27, 69 and 216 ppb in the residual SBF solutions for ES-2ZBG, -4ZBG and -6ZBG, respectively. This suggests that the electrospun PCL/ZBG bioscaffold with Zn addition may exhibit antibacterial ability in addition to their superior bioactivity. It has been reported by Oh et al. [35] that zinc-added bioactive glass exhibited, not only the antibacterial ability, but also ALP activity. However, the high concentration of the zinc-doped bioactive glass may induce cytotoxicity and inhibit bone regeneration [36]. Further investigations concerning the bone regeneration using the PCL/ZBG bioscaffolds are in progress and will be addressed elsewhere.

**Figure 11:**
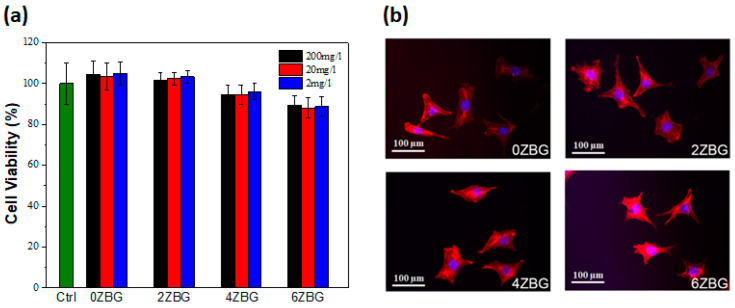
The biocompatibility of various concentrations of extracts from ES-ZBG in (**a**) murine fibroblast L929 cells and (**b**) the cell morphology and cytoskeleton integrity of L929 cells. Please note that the ES-ZBG bioscaffolds were abbreviated as ZBG in the figures, respectively.

**Figure 12:**
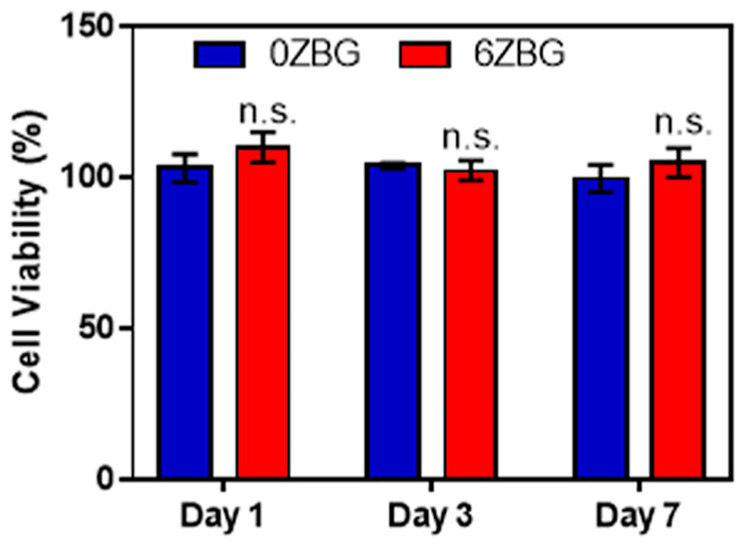
The long-term biocompatibility effects of ES-0ZBG and ES-6ZBG in MG-63 human osteoblast-like cells. ES-0ZBG and ES-6ZBG were abbreviated as 0ZBG and 6ZBG, respectively. No significant differences between 0ZBG and 6ZBG are abbreviated as “n.s.” in the figure.

Biocompatibility and antibacterial performance should be investigated before practical clinical application [34,37,38]. According to the ISO-10993-5 guideline [38], the ZBG extract (with three different concentrations of 200, 20 and 2 mg/L) did not cause significant cell number diminution in murine fibroblast L929 cells (Figure 11a) after an assessment using the CCK-8 viability assay. As shown in Figure 11a, the cell viability slightly decreased with the increasing amount of zinc addition, whereas all ES-ZBG exhibited a cell viability higher than 88%, which met the 70% minimum ISO-10993-5 requirement [38]. In addition, the different percentages of zinc with ES-ZBG did not affect cell morphology and cytoskeleton structure in the L929 cells (Figure 11b) indicating that the ES-ZBG bioscaffolds did not exhibit cytotoxicity. The long-term biocompatibility of the selected ES-0ZBG and ES-6ZBG were evaluated using MG63 human osteoblast-like cells after various durations (1, 3 and 7 days). As shown in Figure 12, there were no significant differences in cell viabilities between the control ES-0ZBG and ES-6ZBG, indicating that the antibacterial modification did not cause cytotoxicity nor interfere with the osteoblast activity in the subsequent bone formation ability of BGs, even in long-term culture conditions.

**Figure 13:**
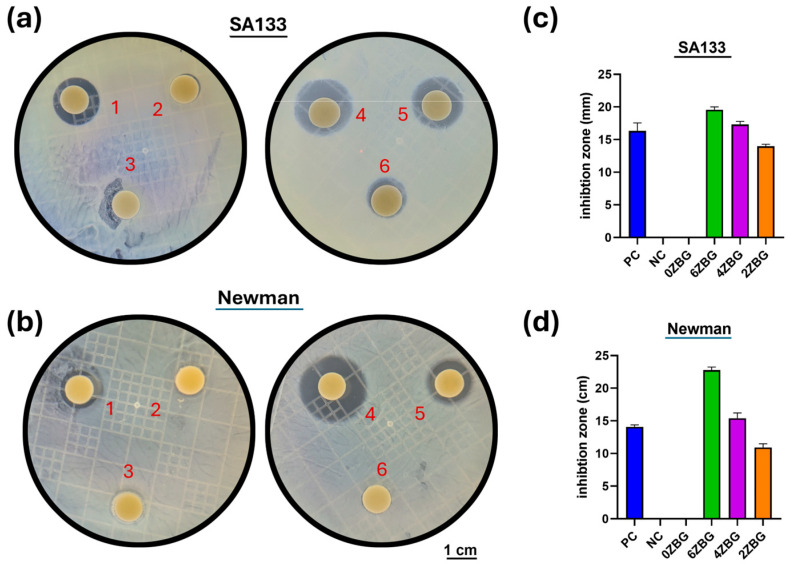
The disk diffusion assay of various ES-ZBGs against two gram-positive *S. aureus* bacteria strains, (**a**) SA133 and (**b**) Newman. 1: P.C., 2: N.C., 3: 0ZBG, 4: 6ZBG, 5: 4ZBG and 6: 2ZBG. Corresponding inhibition zone diameters for those shown in (**a**,**b**) were measured and shown in (**c**) SA133 and (**d**) Newman, respectively.

Figure 13 shows the antibacterial performances against two *S. aureus* bacteria (SA133 and Newman) for ES-ZBG bioscaffolds. It can be observed that the positive control (a disk with antibiotic 30 μg kanamycin) and ES-ZBG exhibited an inhibition zone against SA133 (Figure 13a) and Newman strains (Figure 13b), respectively. As shown in Figure 13c, the average diameter of the inhibition zone against SA133 was 16.3 ± 1.0 mm for position control and was 19.6 ± 0.4 mm, 17.3 ± 0.4 mm and 14.0 ± 0.3 mm for ES-6ZBG, ES-4ZBG and ES-2ZBG, respectively. ES-4ZBG exhibited a slightly larger inhibition zone compared to that of the positive control and antibacterial performance against SA133 increased with the increasing amount of zinc addition. A similar trend was exhibited for the Newman strain (Figure 13d). The inhibition zone was 14.1 ± 0.3 mm, 22.8 ± 0.4 mm, 15.4 ± 0.7 mm, and 10.9 ± 0.5 mm for positive control, ES-6ZBG, ES-4ZBG, and ES-2ZBG, respectively. This suggests that the ES-4ZBG and ES-6ZBG bioscaffolds exhibited a sustained and superior antibacterial ability compared to the chemical antibiotic kanamycin.

## 4. Conclusions

Zinc-doped (0–6 wt.%) bioactive glass powder was prepared via the sol–gel process, followed by calcination at 700 °C for 3 h. ZBG powder exhibited a mixture of dicalcium silicate and an amorphous matrix. By immersing the ZBG powder in SBF for 7 days, the formation of coral-like hydroxyl apatite was observed and displayed superior bioactivity.

Electrospun PCL/ZBG (i.e., ES-ZBG) composite bioscaffolds with an optimal amount of 0.8 g ZBG addition were biocompatible. After immersing the ES-ZBG bioscaffolds in SBF solution, continuous hydroxyl apatite formation was observed, indicating excellent bioactivity. Furthermore, all ES-ZBG exhibited an inhibition zone against two *S. aureus* bacteria strains, SA133 and Newman and the antibacterial performance increased with increasing Zn^2+^ concentration in ES-ZBG bioscaffolds. The ES-ZBG composite bioscaffolds were biocompatible, antibacterial, highly bioactive and suitable for tissue engineering applications.

## Figures and Tables

**Table 1:** Sample codes for zinc-added bioactive glass powder after calcination at 700 °C for 3 h.

Sample Code	SiO_2_ (wt.%)	CaO (wt.%)	P_2_O_5_ (wt.%)	ZnO (wt.%)
0ZBG	58	33	9	0
2ZBG	58	31	9	2
4ZBG	58	29	9	4
6ZBG	58	27	9	6

## Data Availability

The original contributions presented in the study are included in the article, further inquiries can be directed to the corresponding authors.
